# Supplementary material for: Knockdown of Quinolinate Phosphoribosyltransferase Results in Decreased Salicylic Acid-Mediated Pathogen Resistance in Arabidopsis thaliana
Source: Int J Mol Sci. 2021 Aug 6;22(16):8484. doi: 10.3390/ijms22168484 (PMC8395217; doi:10.3390/ijms22168484)
Supplement: Supplementary file 1 [file ijms-22-08484-s001.zip › ijms-1184089-supplementary.pdf]

Table S1. List of primers used in the study.

Name	Sequence (5'-3')	Technique
RiQPRT-Fwd1	TCATTTGGAGAGGACACGCTCGAGGCCTCCT TCACACCCTACTTAC	pHY6 construction
RiQPRT-Rev1	AGCTGGGGTACCGAATTCCTCGAGTGCAATT CCACTCATCCTCTG	pHY6 construction
RiQPRT-Fwd2	ACTCATTAAAGCAGGACTCTAGAGCCTCCTTC ACACCCTACTTAC	pHY6 construction
RiQPRT-Rev2	ATCGATAAGCTTGGATCCTCTAGATGCAATTC CACTCATCCTCTG	pHY6 construction
q-QPRT-Fwd	TTGTCGCCTCAATTCTACGCAAT	qRT-PCR
q-QPR-Rev	ACATCTCCTGTATGACCAGCATCT	qRT-PCR
q-AOX1-Fwd	ACATCTGCTTGGATATGGACTA	qRT-PCR
q-AOX1-Rev	TCGTCGGAGCTCTAGTCCAT	qRT-PCR
q-FER1-Fwd	AACGCACTCTCGTCTTTCAC	qRT-PCR
q-FER1-Rev	ATCGGCTTTCTTCACCTCTT	qRT-PCR
q-GSTU24-Fwd	GGCGAGTATGTTTGGGATG	qRT-PCR
q-GSTU24-Rev	TTCATCTCGAGGAGCAAGG	qRT-PCR
q-OXI1-Fwd	GTTGAGGAAATCAAGGGTCATG	qRT-PCR
q-OXI1-Rev	TGGACGATATTCTCCACATCC	qRT-PCR
q-ICS1-Fwd	TTGGTGGCGAGGAGAGTG	qRT-PCR
q-ICS1-Rev	CTTCCAGCTACTATCCCTGTCC	qRT-PCR
q-PR1-Fwd	AGGCTAACTACAACTACGCTGCG	qRT-PCR
q-PR1-Rev	GCTTCTCGTTCACATAATTCCCAC	qRT-PCR
q-PR5-Fwd	ATCGGGAGATTGCAAATACG	qRT-PCR
q-PR5-Rev	ATGACCTTAAGCATGTCGGG	qRT-PCR

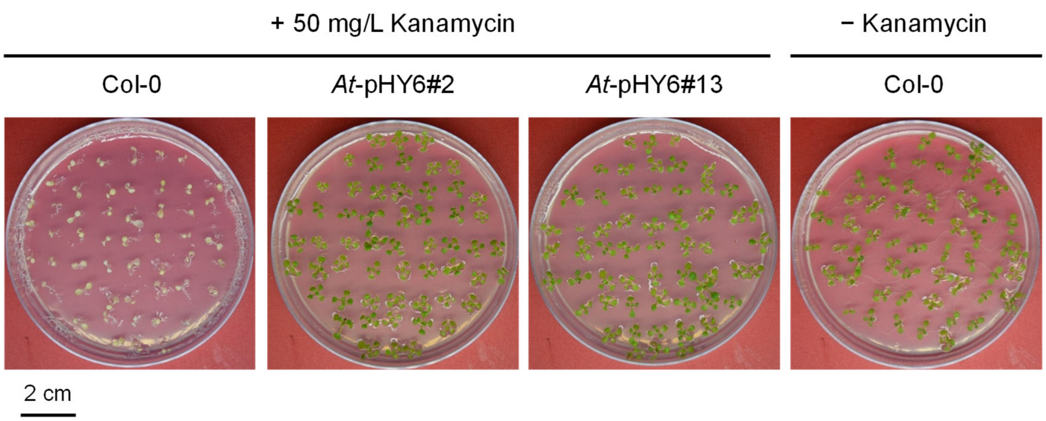

Figure S1. Seed assays confirming the homozygous status of the *QPRT* RNAi plants. Seeds of Col-0 and the *QPRT* RNAi plants were germinated on MS medium containing 50 mg·L^{-1} kanamycin (Col-0, *At*-pHY6#2, *At*-pHY6#13) or antibiotic-free medium (Col-0).

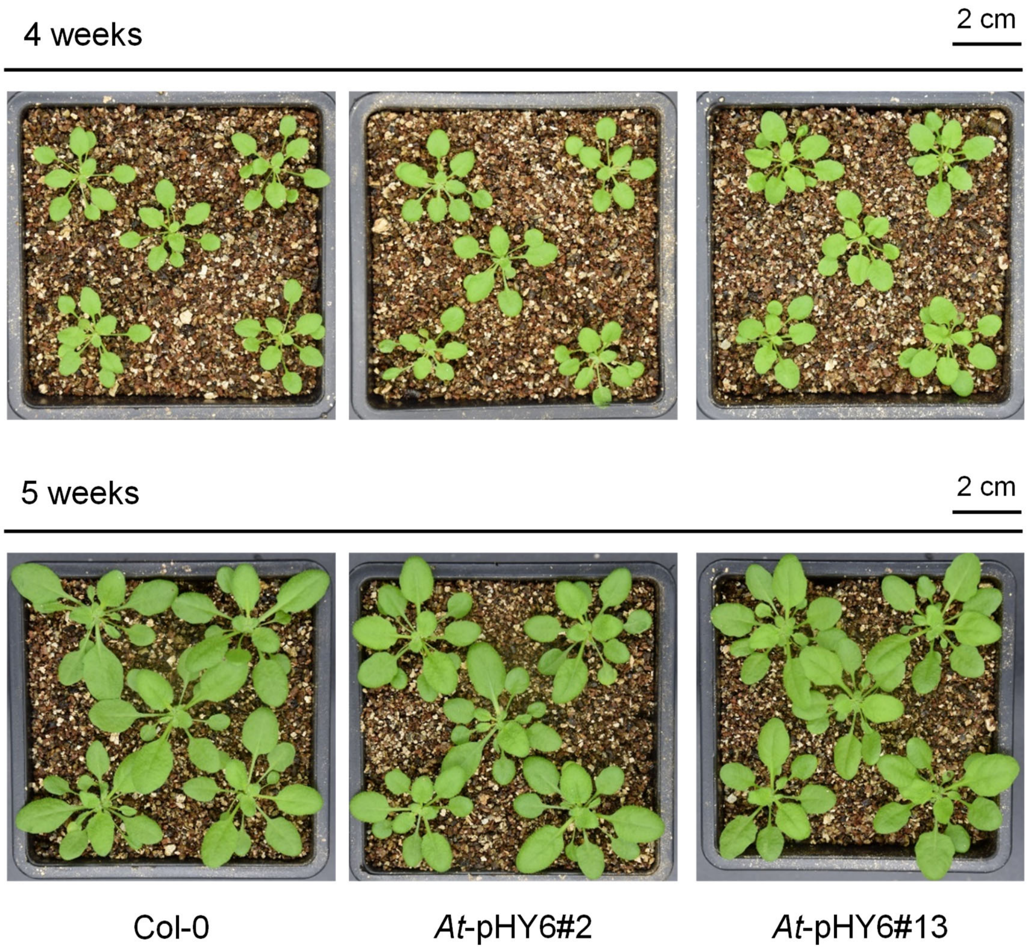

Figure S2. Knockdown of *QPRT* expression by RNAi did not affect plant growth under optimal growth condition. Representative photographs of Col-0 and *QPRT* RNAi plants.

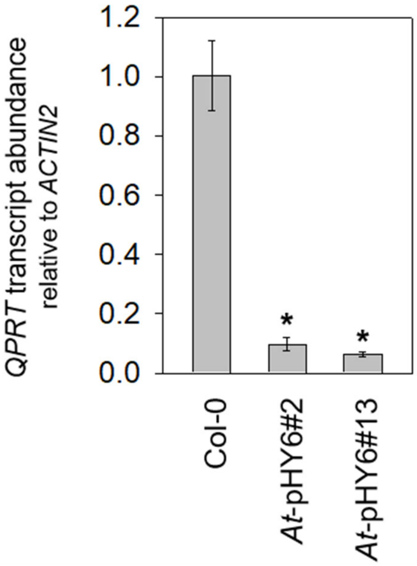

Figure S3

Figure S3. *QPRT* mRNA levels at 48 hpi. Means ± SE of three repetitions are shown. Asterisks indicate that values are significantly different from those of Col-0 at $^{*}P < 0.05$.
